# ﻿A new species of the genus *Separatatus* Chen & Wu (Hymenoptera, Braconidae, Alysiinae) from South Korea

**DOI:** 10.3897/zookeys.1097.82860

**Published:** 2022-04-29

**Authors:** Ju-Hyoeng Sohn, Cornelis van Achterberg, Sangjin Kim, Jongok Lim, Hyojoong Kim

**Affiliations:** 1 Animal Systematics Lab., Department of Biological Science, Kunsan National University, Gunsan, 54150, Republic of Korea; 2 State Key Laboratory of Rice Biology and Ministry of Agriculture, Zhejiang University, Hangzhou, 310058, China; 3 Key Lab of Agricultural Entomology, Institute of Insect Science, Zhejiang University, Hangzhou, 310058, China; 4 Department of Life and Environmental Sciences, Wonkwang University, Iksan, JB 54538, Republic of Korea

**Keywords:** *COI* barcode, cyclostome, koinobiont, natural enemy, parasitoid wasp, systematics, taxonomy

## Abstract

*Separatatusmegagnathus***sp. nov.** is recorded as new to science from South Korea. Due to this record, the genus *Separatatus* Chen & Wu, 1994 (Braconidae: Alysiinae) is recognized for the first time from South Korea. The genus and species are described and illustrated herein plus an identification key including the Korean new species is provided. In addition, the DNA barcode region of the mitochondrial cytochrome c oxidase subunit I (*COI*) has been analyzed for the new species.

## ﻿Introduction

The subfamily Alysiinae is a relatively large taxon among the family Braconidae, which contains over 2450 valid species and is subdivided into two tribes, Alysiini with 76 genera and Dacnusini with 31 genera (Yu et al. 2016; Peris-Felipo and Belokobylskij 2020). There are 180 species of 21 genera listed in the National Species List of South Korea, South Korea (NIBR 2020). It is known that Alysiini includes mostly koinobiont endoparasitoids of cyclorrhaphous dipteran larvae, which are recognized by using their mandible (usually with 3–4 teeth or lobes) to break open the puparium of the host (Wharton 1977). Some species of Alysiinae are utilized for biological control (Abd-Rabou 2006).

The genus *Separatatus* Chen & Wu, 1994 is a small group of Alysiinae, which includes five Oriental or East Palaearctic species (Yu et al. 2016; Zhu et al. 2017; Zhang et al. 2020). This genus is easily characterized by the rugose second metasomal tergite, robust mandible and metanotum slightly or not protruding dorsally (Zhang et al. 2020). Chen and Wu (1994) created the genus *Separatatus* and described *Separatatuscarinatus* Chen & Wu, 1994 as new species from China. *Bobekioidessinicus*, which had been described by Zheng et al. (2013) from China, was transferred to *Separatatus* by Zhu et al. (2017). Zhu et al. (2017) also described a new species, *S.parallelus*, from China. Yao et al. (2018) transferred *Phasmidiastamalaysiae* Fischer, 2006 to *Separatatus*, and added a new species, *S.xuexincheni*, from Thailand. Recently, Zhang et al. (2020) described a new species, *S.yinshani*, from China.

In this study, *Separatatusmegagnathus* sp. nov. is recorded as new to science from South Korea. Simultaneously, the genus *Separatatus* Chen & Wu, 1994 (Braconidae: Alysiinae) is recognized for the first time from South Korea. We descried the morphological characters and the barcoding sequences of the *COI* region of this new species. A description, diagnosis and photographs of the diagnostic characters are also provided.

## ﻿Materials and methods

The holotype was collected with a Malaise trap in South Korea at the DMZ Botanical Garden, Mandae-ri, Haean-myeon, Yanggu-gun, Gangwon-do. Sorting and preparation were done at the Animal Systematics Lab. (**ASL**), Department of Biological Science, Kunsan National University (**KSNU**) at Gunsan, South Korea. Zhu et al. (2017), Yao et al. (2018) and Zhang et al. (2020) were followed for the morphological identification. Morphological characters were observed with a Leica M205C stereo microscope. The Taxapad database (Yu et al. 2016) was used for checking valid species and references. We followed the terminology of Wharton (2002) and van Achterberg (1993). The holotype is deposited in the Insect Museum, Department of Biological Science, KSNU.

A LEICA DMC2900 digital camera and a LEICA M205 C microscope (Leica Geosystems AG, Wetzlar, Germany) were used for photography and several pictures being taken for each height using multifocusing technology. LAS V4.11 (Leica Geosystems AG, Wetzlar, Germany) and HeliconFocus 7 (Helicon Soft, Kharkiv, Ukraine) software were used for image stacking. After stacking, plates were created using Adobe Photoshop CS6.

Extraction of DNA was done in ASL, KSNU. Whole genomic DNA was extracted from the specimen by using a DNeasy Blood & Tissue kit (QIAGEN Inc., Dusseldorf, Germany) following the manufacturer’s protocol. In order to conserve the morphologically complete voucher specimen, DNA extraction was slightly modified from the ‘non-destructive method’ of Favret (2005) and the ‘freezing method’ of Yaakop et al. (2009). In the original protocol, the sample was crushed or wounded, and then soaked with 180 μl of buffer ATL + 20 μl of proteinase, following by three hours over incubation at 55 °C. In our slightly modified DNA extraction methods, samples were soaked with 180 μl of buffer ATL + 20 μl of proteinase K without destroying the sample, followed by 10 minutes incubation at 55 °C and then kept in a freezer at -22 °C overnight. After that, the general protocol was used for the remaining steps. The primerset of LCO-1490 (5’-GGTCAACAAATCATAAAGATATTGG-3’) and HCO-2198 (5’-TAAACTTCAGGGTGACCAAAAAATCA-3’) (Folmer et al. 1994) was used to amplify approximately 658 bp as the partial front region of the *COI*. The polymerase chain reaction (PCR) products were amplified by using AccuPowerH PCR PreMix (BIONEER, Corp., Daejeon) in 20 μl reaction mixtures containing 0.4 μM of each primer, 20 μM of the dNTPs, 20 μM of the MgCl_2_, and 0.05 μg of the genomic DNA template. PCR amplification was performed using a GS1 thermo-cycler (Gene Technologies, Ltd., Essex, U.K) according to the following procedure: initial denaturation at 95 °C for 5 min, followed by 34 cycles at 94 °C for 35 s; an annealing temperature of 48 °C for 25 s; an extension at 72 °C for 45 s, and a final extension at 72 °C for 5 min. The PCR products were visualized by electrophoresis on a 1.5% agarose gel. A single band was observed, purified using a QIAquick PCR purification kit (QIAGEN, Inc., Milan, Italy), and then sequenced directly using an automated sequencer (ABI Prism 3730 XL DNA Analyzer, ABI, Waltham, MA, USA) at Macrogen Inc. (Seoul, South Korea).

## ﻿Results and discussion

A total of 620 bp of the COI fragments were sequenced from *Separatatusmegagnathus* sp. nov. which was deposited in GenBank (accession number MZ717197). Unfortunately, it could not be used to measure genetic distance between related taxa because there is no sequence of a congeneric species in GenBank. However, the sequence will facilitate the recognition of the new species if other sequences of *Separatatus* are added in future.

### ﻿ Separatatus

Taxon classificationAnimaliaHymenopteraBraconidae

Chen & Wu, 1994

74D68BAB-2E41-519D-8044-501A527E52E7

Separatatus Chen & Wu, 1994: 132. Type species: Separatatuscarinatus Chen & Wu, 1994.

#### Diagnosis.

**Figure 1.**
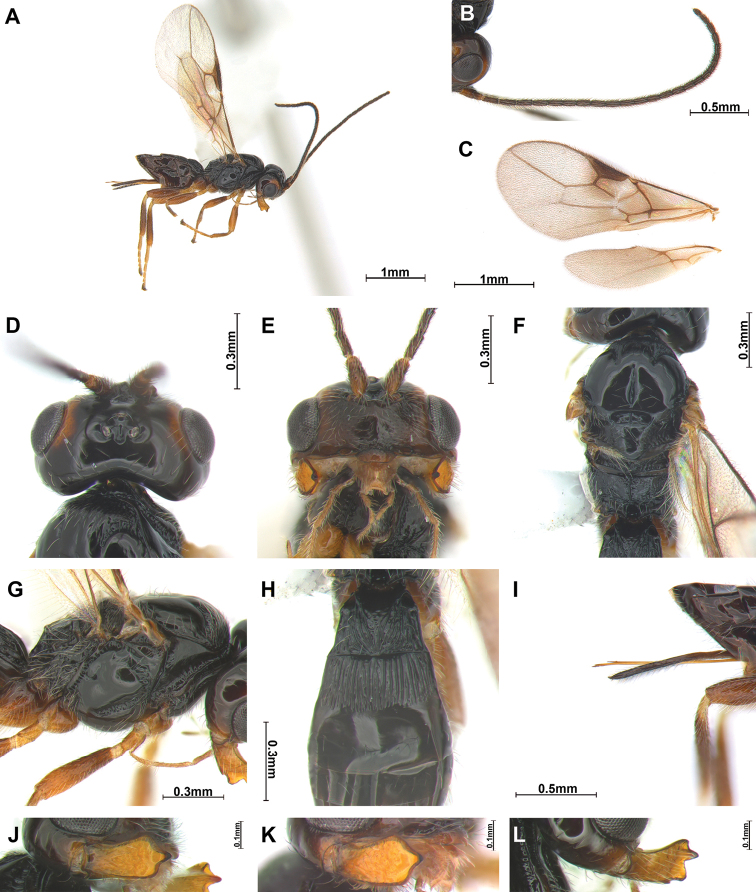
*Separatatusmegagnathus* sp. nov., ♀ **A** habitus, lateral view **B** antennae **C** wings **D** head, dorsal view **E** head, front view **F** mesosoma, dorsal view **G** mesosoma, lateral view **H** anterior half of metasoma, dorsal view **I** ovipositor sheath, lateral view **J** mandible, lateral view **K** mandible, antero-lateral view **L** mandible with teeth. Scale bars: 1 mm (**A, C**); 0.5 mm (**B, I**); 0.3 mm (**D–H**); 0.1 mm(**J–L**)

Antenna 1.0–1.3 times longer than body; first flagellomere slightly shorter than second (Fig. 1B), face with setae (Fig. 1E), eye slightly oval and glabrous; clypeus semicircular; labrum long and triangularly shape, mandible with 3–4 teeth or lobes (Fig. 1J), fourth ventral tooth (if present) small; maxillary palp with 6 segments; notauli present on anterior third of mesoscutum; medio-posterior depression distinct, round or longitudinal; scutellar sulcus distinct; fore wing (Fig. 1C) vein 2-SR slightly bent, vein 2-SR slightly shorter than vein 3-SR; vein 2-SR+M not sclerotized; hind wing vein 1-M longer than vein 1r-m; second tergite rugose, longer than first (Fig. 1H); ovipositor sheath slightly shorter than metasoma; tarsal claws slender.

#### Biology.

Unknown.

#### Distribution.

Oriental and Palaearctic region.

### ﻿Key to species of subgenus Separatatus

**Table:** 

1	Basal part of pterostigma long and parallel-sided; vein r close to apex of pterostigma; [vein 3-SR of fore wing about 2.9 times longer than vein r; hind femur ca 2.7 times longer than wide]; China (Yunnan, Hainan)	***S.parallelus* Zhu, van Achterberg & Chen, 2017**
–	Basal part of pterostigma comparatively short and elliptical; vein r more removed from apex of pterostigma; [vein 3-SR of fore wing ca 3.0 times longer than vein r, also pterostigma more robust]	**2**
2	Mandible hardly emarginated between first and second teeth of mandible (Figs 1J-L); ventral lobe of mandible small (Figs 1J-L); body black or blackish; precoxal sulcus present anteriorly (Fig. 1G); legs partly brown or dark brown (Fig. 1A); [medio-posterior depression of mesoscutum elongate and about half as long as mesoscutum medially; S. Korea]	***S.megagnathus* Sohn & van Achterberg, sp. nov.**
–	Emargination between first and second teeth of mandible distinct; ventral lobe of mandible medium-sized; body reddish brown or yellowish brown; precoxal sulcus absent anteriorly; legs yellow	**3**
3	Vein r-m of fore wing weakly inclivous; [hind femur of ♀ rather inflated, ca 3.0 times longer than wide]; China (Yunnan)	***S.carinatus* Chen & Wu, 1994**
–	Vein r-m of fore wing strongly inclivous	**4**
4	Head and fourth antennal segment yellow; hind femur of ♀ ca 3.5 times longer than wide; West Malaysia	***S.malaysiae* (Fischer, 2006)**
­–	Head and fourth antennal segment mainly dark brown; hind femur of ♀ ca 2.6 times longer than wide; Thailand	***S.xuexincheni* Yao, 2018**

### ﻿ Separatatus megagnathus

Taxon classificationAnimaliaHymenopteraBraconidae

Sohn & van Achterberg sp. nov.

2D3BA588-3BAA-5E5D-94D5-4E38EE1E1F0E

http://zoobank.org/0814AA28-EF2F-4AD8-B33A-5CE461BC36DD

Fig. 1A–L

#### Type material.

***Holotype***, ♀ (KNA), **South Korea**, DMZ Botanical Garden, Mandae-ri, Haean-myeon, Yanggu-gun, Gangwon-do, 38°15'09.3"N,128°06'40.6"E, 20.VI.–4.VII.2017, Shin & Kim leg. GenBank accession number MZ717197.

#### Comparative diagnosis.

The new species belongs to the subgenus Separatatus Chen & Wu (Zhang et al. 2020). It runs in the key by Yao et al. (2018) to *S.malaysiae* because of the less developed areola of the propodeum and distinctly postfurcal vein m-cu of the fore wing. Both species can be separated as follows:

**Table:** 

1	Emargination between first and second teeth of mandible absent or nearly so (Fig. 1L); medio-posterior depression of mesoscutum elongate and about half as long as mesoscutum medially (Fig. 1F); ventral (= fourth) lobe of mandible small (Fig. 1L); body black or blackish; precoxal sulcus present anteriorly (Fig. 1G); legs partly brown or dark brown (Fig. 1A); S. Korea	***S.megagnathus* Sohn & van Achterberg, sp. nov.**
–	Incision between first and second teeth of mandible present; medio-posterior depression of mesoscutum round and small; ventral lobe of mandible medium-sized; body reddish brown or yellowish brown; precoxal sulcus absent anteriorly; legs yellow; W. Malaysia	***S.malaysiae* (Fischer, 2006)**

#### Description.

♀: length of body in lateral view 2.5 mm, length of antenna 2.6 mm, and length of fore wing 2.8 mm. ***Colour*.** Body entirely dark brown; head in dorsal view entirely black, in anterior view reddish brown, around eye brown, antenna brown, mandible orangish brown and apically dark brown. ***Head*.** Head (Fig. 1D) width 2.2 times median length in dorsal view. Antenna (Fig. 1B) as long as body, 25 segmented. First flagellomere 0.9 times as long as second. Eye slightly oval and glabrous, 1.1 times as long as wide in lateral view. Width of face (Fig. 1E) 2.2 times its height from ventral rim of antennal sockets to upper margin of clypeus; face with long setae and smooth. Eye in dorsal view 1.2 times as long as temple. Ocello-ocular line (OOL) 4.2 times longer than diameter of anterior ocellus; OOL:antero-posterior ocellar line (AOL):postero-ocellar line (POL) = 19:6:8. Vertex smooth and polish with groove. Mandible (Fig. 1J) with four teeth and setae; dorsal tooth large and lobe-shaped, small incision between first and second teeth; ventral (fourth) tooth lobe-shaped, middle of tooth curved down; second tooth relatively narrow and sharp with dark brown tip and separated from first tooth by incision in lateral view (Fig. 1L). Medial length of mandible 1.5 times its maximum width. Labrum 3.2 times longer than wide. Maxillary palp 0.7 times longer than mesosoma. ***Mesosoma*.** Mesosoma (Fig. 1G) 1.8 times longer than wide in dorsal view and 1.4 times its height in lateral view; notauli crenulated, not reaching medio-posterior depression; medio-posterior depression distinctly elongated, half as long as mesoscutum medially; scutellar sulcus with two carinae; in lateral view mesopleuron smooth and shiny, precoxal sulcus (Fig. 1F) distinct but absent posteriorly and with 11 crenulae; metapleuron distinctly rugose and with long setae. Propodeum largely smooth, its median carina medium-sized, connected to irregular transverse carina (Fig. 1F); posterior areola incomplete; in lateral view propodeum curved dorsally, with submedian corner. ***Wings*.** Fore wing (Fig. 1C) 2.3 times longer than wide; pterostigma long and rather broad, 3.8 times longer than wide; base of vein 1-R1 narrow; vein r of fore wing 5.0 times longer than wide, 0.6 times width of pterostigma and arising from its basal 0.6; vein 1-M and vein 1-SR+M slightly bent; vein 2-SR+M and r-m not sclerotized; vein r-m inclivous; vein 2-SR:vein r:vein 3-SR = 10:3:11; first subdiscal cell of fore wing 2.5 times longer than wide medially; vein m-cu distinctly postfurcal. Hind wing 4.1 times longer than wide; vein M+CU slightly bent; vein M+CU:1-M:1r-m = 11:6:3. ***Legs*.** Hind coxa smooth and 1.1 times longer than trochanter; hind femur 0.8 times as long as hind tibia and 7.1 times longer than wide; hind tibia as long as hind tarsus. ***Metasoma*.** First tergite widened posteriorly, striate and narrow, 0.8 times longer than its apical width; first tergite 0.6 times longer than second. Setose part of ovipositor sheath (Fig. 1I) 0.8 times longer than mesosoma, as long as hind tibia and with long setae. **Male.** Unknown.

#### Distribution.

South Korea.

#### Etymology.

From “megas” (Greek for large) and “gnathos” (Greek for jaw) because of the large mandible.

## Supplementary Material

XML Treatment for Separatatus

XML Treatment for Separatatus megagnathus
